# Randomized, Double-blind, Placebo-controlled Study of a Multispecies Probiotic Mixture in Nonalcoholic Fatty Liver Disease

**DOI:** 10.1038/s41598-019-42059-3

**Published:** 2019-04-05

**Authors:** Sang Bong Ahn, Dae Won Jun, Bo-Kyeong Kang, Jong Hyun Lim, Sanghyun Lim, Myung-Jun Chung

**Affiliations:** 1 Department of Internal Medicine, Eulji University School of Medicine, Seoul, Korea; 2 Department of Internal Medicine, Hanyang University School of Medicine, Hanyang University Medical Center, Seoul, Korea; 3 Department of Radiology, Hanyang University School of Medicine, Hanyang University Medical Center, Seoul, Korea; 4 R&D center Microbiome, Cell Biotech, Gyeongi, Korea

## Abstract

The intestinal microbiota is closely associated with the development of obesity and nonalcoholic fatty liver disease (NAFLD). This study investigated the effects of probiotic treatment on visceral fat area (VFA) and intrahepatic fat (IHF) fraction in NAFLD. Sixty-eight obese NAFLD patients (>5% proton density fat fraction [PDFF] on magnetic resonance imaging [MRI]) were randomized to probiotic and placebo groups for 12 weeks. The probiotic mixture included 6 bacterial species. VFA and IHF were measured using the MRI-PDFF technique. Body weight and total body fat were reduced in the probiotic group but not in the placebo group. The mean IHF fraction was reduced after 12 weeks of treatment in the probiotic group compared to that at baseline (from 16.3 ± 15.0% to 14.1 ± 7.7%, p = 0.032) but was not reduced in the placebo group. The decrease in IHF (mean difference: −2.61%, p = 0.012) was also greater in the probiotic group than in the placebo group. Reduction of triglyceride was greater in the probiotic treatment group than in the placebo group (mean difference: −34.0 mg/dl, p = 0.0033). However, the changes in IHF percentage and triglyceride levels were not different between placebo and control groups after adjusting for changes in body weight. Treatment with probiotics for 12 weeks resulted in significant reduction in IHF and body weight in obese NAFLD patients.

## Introduction

With an increase in the prevalence of obesity, nonalcoholic fatty liver disease (NAFLD) has increased in its societal significance. The intestinal microbiota plays an important role in the development and progression of obesity and NAFLD. Recent studies have demonstrated the significance of aberrations in the gut microbiota composition in NAFLD^1^. The beneficial effects of probiotics have been confirmed in several experimental studies^2–4^.

Randomized controlled studies have evaluated the efficacy of probiotics in adult patients with NAFLD^5–11^. However, most involved the administration of prebiotics along with probiotics^12,13^, most were open-label studies^6,7,10,11^, and only 2 were double-blind, randomized controlled studies that explored the effects of probiotic administration in NAFLD patients^5,9^. Moreover, the latter 2 studies used alanine transaminase (ALT) and aspartate transaminase (AST) as primary endpoints to assess improvement in fatty liver and did not evaluate intrahepatic fat (IHF), visceral fat area (VFA), or the degree of liver fibrosis. No reported study has quantitatively assessed changes in IHF and hepatic fibrosis after administration of probiotics. In addition, data are lacking on changes in microbiome composition and metabolism after probiotic treatment.

This study administered a mixture of 6 probiotic agents (*Lactobacillus acidophilus, L. rhamnosus, L. paracasei, Pediococcus pentosaceus, Bifidobacterium lactis*, and *B. breve*) most commonly used in the study of obesity and fatty liver-related diseases. We aimed to investigate the effects of probiotic administration on VFA and IHF in obese NAFLD patients.

## Results

### Baseline characteristics

**Figure 1:**
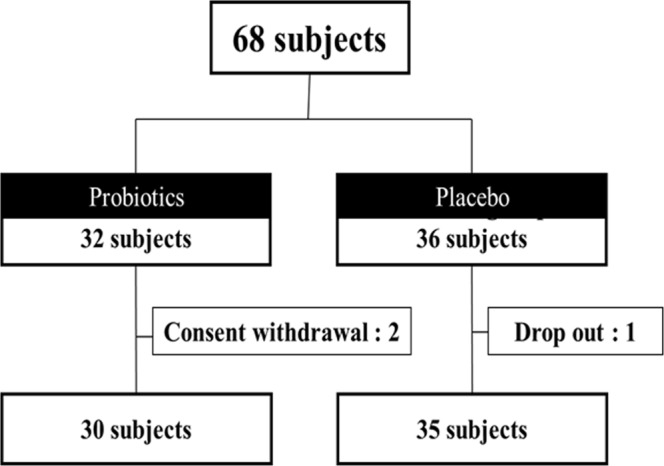
Consort flow chart.

Sixty-eight obese NAFLD patients were randomized into probiotic and placebo groups. Thirty (93.8%) and 35 patients (97.2%) completed the study in the probiotic and placebo groups, respectively (Fig. 1). The mean age was 43.2 years, and 32 men and 33 women participated (Table 1). The mean proton density fat fraction (PDFF) on magnetic resonance imaging (MRI-PDFF) was 16.2%. IHF, body mass index (BMI), biochemical parameters, abdominal total fat area, and VFA were comparable in both groups.

**Table 1:** Baseline subject characteristics.

	Total	Probiotic (n = 30)	Placebo (n = 35)	*p
Age (years)	43.32 ± 12.9	41.7 ± 12.49	44.71 ± 13.31	0.36
Male patients (%)	33 (48.5%)	15/32 (47%)	18/36 (50%)	0.81
Weight (kg)	82.23 (79.6)	81.79 ± 13.56	82.61 (79.3)	0.90
BMI (kg/m^2^)	30.08 (29)	30.05 (28.8)	30.11 (29.1)	0.94
Cholesterol (mg/dl)	198.8 ± 37.5	201.27 ± 33.78	196.69 ± 40.83	0.62
Triglyceride (mg/dl)	179.5 (147)	187.37 (152)	172.8 (140)	0.20
HDL-Cholesterol	47.0 (43)	46.6 (43)	47.371 (48)	0.64
Glucose (mg/dl)	105.87 (93)	112.7 (92.5)	100.02 (93)	0.82
AST (U/L)	32.72 (26)	33.57 (27)	32 (23)	0.74
ALT (U/L)	41.48 (31)	41.13 (31.5)	41.77 (28)	0.68
Insulin (μU/ml)	14.75 (10.2)	17.05 (10.15)	12.77 (10.2)	0.70
Total fat area (cm^2^)	34656.4 (33058.2)	33734.4 (31505.6)	35446.7 ± 9108.9	0.32
Visceral fat area (cm^2^)	15268.3 (14846.8)	14612.4 (14793.4)	15830.4 ± 5865.65	0.36
Muscle mass (kg)	48.4 (46.7)	48.12 ± 10.54	48.73 ± 11.80	0.83
Total fat mass (kg)	30.91 (29.1)	30.78 (29.5)	31.03 (28.7)	0.74
Total fat (%)	37.57 ± 7.12	37.89 ± 6.72	37.3 ± 7.53	0.74
Visceral fat (grade)	14.18 ± 4.18	14.07 ± 3.78	14.28 ± 4.54	0.84
Waist-hip ratio	0.95 ± 0.056	0.95 ± 0.057	0.96 ± 0.05	0.42
CAP score	311.43 (313)	313.67 (319.5)	309.51 ± 47.60	0.30
Liver fat fraction (%)	15.00 (13.9)	16.25 (14.95)	13.93 (11.6)	0.25

Mean ± standard deviation is presented for normally distributed variables, and median (interquartile range) for non-normally distributed variables. BMI, body mass index; HDL, high-density lipoprotein; AST, aspartate transaminase; ALT, alanine transaminase; CAP, controlled attenuation parameter; *p < 0.05 by t-test and chi-square test.

### Body weight and body fat composition

**Figure 2:**
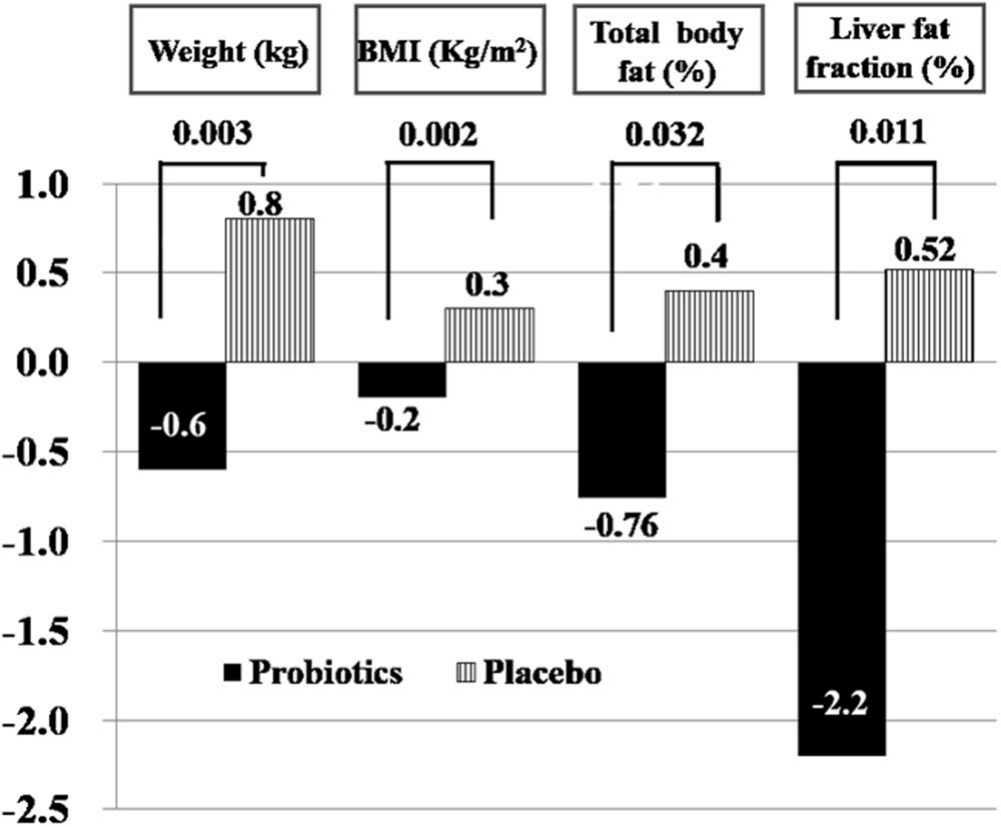
Changes in weight and body fat according to treatment.

Patients in the probiotic group had greater loss in body weight (mean difference: −1.35 kg, p = 0.0029), and BMI (mean difference: −0.48 kg/m^2^, p = 0.0024) than the placebo group (Table 2). Total fat mass (kg) (mean difference: −1.25 kg, p = 0.0028), total body fat percent (%) (mean difference: −0.9%, p = 0.0032), and visceral fat grade (mean difference: −1.12%, p = 0.0029) measured with bioimpedance analysis were decreased in the probiotic group relative to those in the placebo group (Fig. 2). However, changes in VFA and abdominal fat area measured with MRI-PDFF were not different between the 2 groups.

**Table 2:** Change in weight and body fat according to treatment.

	Probiotic (n = 30)			Placebo (n = 35)			Difference (p-value)
	Baseline	3 months	*P	Baseline	3 months	*P	
Weight (kg)	81.8 ± 13.6	81.2 ± 13.5	0.072	**82.6 (79.3)**	**83.4 (79.5)**	**0.012**	**−1.35 (0.0029)**
BMI (kg/m^2^)	**30.0 ± 3.6**	**29.8 ± 3.6**	**0.047**	**30.1 (29.1)**	**30.4 (28.8)**	**0.024**	**−0.48 (0.0024)**
Visceral fat (cm^2^)	146.1 (14.7)	144.3 (14.4)	0.730	158.35 ± 58.6	158.2 ± 59.5	0.992	−169.5 (0.7590)
Total fat mass (kg)	30.8(29.5)	29.95 ± 6.4	0.104	31.0 (28.7)	31.5 (30)	0.07	**−1.25 (0.0282)**
Total body fat (%)	37.9 ± 6.7	37.1 ± 6.3	0.146	37.3 ± 7.53	37.7 ± 7.31	0.260	**−0.90 (0.0324)**
Visceral fat grade	14.0 ± 3.7	13.6 ± 3.4	0.192	14.3 ± 4.55	14.9 ± 4.8	0.092	**−1.12 (0.0297)**
Right liver FF (%)	17.3 (16.1)	15.1 ± 7.7	0.053	14.7 (13.3)	15.3 (12.8)	0.272	**−2.63 (0.0284)**
Left liver FF (%)	**15.2 (12.9)**	**13.1 ± 7.9**	**0.019**	13.1(10.0)	13.6 (10.3)	0.302	−**2.59 (0.0087)**
Whole liver FF (%)	**16.3 (15.0)**	**14.1 ± 7.7**	**0.032**	13.9 (11.6)	14.4 (11.5)	0.255	**−2.61 (0.0129)**
Waist-hip ratio	0.94 ± 0.06	0.95 ± 0.05	0.239	0.96 ± 0.05	0.97 ± 0.06	0.113	−0.001 (0.9317)
CAP (dB/m)	313.4 ± 60.3	315.3 ± 42.3	0.859	310.0 ± 47.0	305.4 ± 47.0	0.569	5.69 (0.626)
Liver stiffness (kPa)	9.65 ± 16.2	6.17 ± 2.50	0.221	6.76 ± 3.42	6.18 ± 2.88	0.142	−3.39 (0.28)

Data are expressed as median (interquartile range) with p values from Wilcoxon signed-rank test or mean difference with p value in parentheses.

BMI: body mass index, FF: fat fraction. Abdominal fat and visceral fat area were measured with magnetic resonance imaging. Total fat mass and total body fat were measured with bio-impedance. *p < 0.05 by paired t-test.

### Intrahepatic fat amount

**Figure 3:**
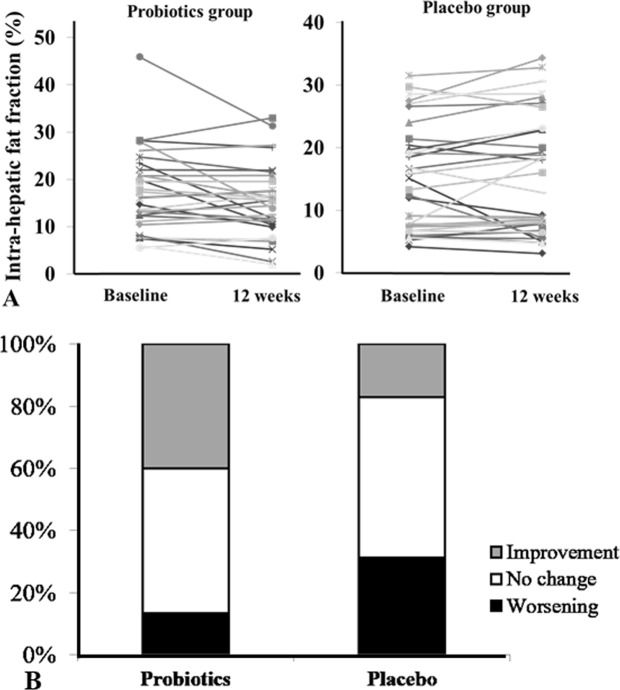
Changes in hepatic fat content in probiotic and placebo groups. (**A**) Individual patient data for hepatic fat content as assessed using MRI-PDFF at baseline and 12 weeks. The probiotic group had a significant reduction in hepatic fat after 12 weeks of treatment. (**B**) In the probiotic group, 40.0% showed a significant reduction in IHF, with no difference in 46.7% and worsening in 13.3%. In the control group, 17.1% showed a significant reduction in IHF, with no difference in 51.4% and worsening in 31.4%. More significant reduction in IHF was shown in the probiotic group than in the control group (40% vs. 17.1%, p = 0.038).

The IHF fraction (16.3 ± 15.0% →14.1 ± 7.7%, p = 0.032) was decreased after 12 weeks of probiotic treatment compared to that at baseline, but was not reduced in the placebo group. The decrease in IHF (mean difference: −2.61%, p = 0.0129) was also greater in the probiotic group than in the placebo group. The decrease in hepatic fat fraction (FF ≥ −1.7% compared to baseline) was greater in the probiotic group than in the placebo group (40% vs. 17.1%, p = 0.038) (Fig. 3). IHF was increased compared to baseline in 13.3% of the probiotic group and 31.4% of the placebo group. The IHF fraction did not change in the placebo group (13.9 ± 11.6% →14.4 ± 11.5%, p = 0.255).

Analysis of covariance was used to adjust for confounding factors such as total caloric intake and weight changes during the experimental period. Changes in IHF amount (FF percent using MRI-PDFF) was not different between placebo and control groups after adjustment for changes in body weight and caloric intake (Table 3).

**Table 3:** Comparison of estimated marginal means of clinical parameters after adjusting for weight and caloric intake using analysis of covariance.

	Adjustment for changes in caloric intake			Adjustment for changes in body weight and caloric intake		
	Probiotic (n = 30)	Placebo (n = 35)	*p	Probiotic (n = 30)	Placebo (n = 35)	*p
Changes of whole liver fat (%)	**2.06 (0.7)**	−**0.51 (0.68)**	**0.014**	1.16 (0.71)	0.25 (0.65)	0.375
Changes of right liver fat (%)	**2.09** (**0.8)**	−**0.51** (**0.72)**	**0.018**	1.13 (0.74)	0.31 (0.68_	0.444
Changes of left liver fat (%)	**2.05 (0.73)**	−**0.50** (**0.67)**	**0.013**	1.20 (0.70)	0.22 (0.64)	0.335
Changes of visceral fat area (cm^2^)	224.3 (406.7)	−40.0 (376.1)	0.637	−56.8 (419.6)	200.9 (385.1)	0.669
Changes of total fat area (cm^2^)	667.9 (637.7)	−906.2 (589.8)	0.077	228.9 (658.1)	−529.9 (604.1)	0.424
Changes of triglyceride (mg/dl)	39.1 (16.2)	−6.24 (15.0)	0.046	31.4 (17.0)	0.30 (15.6)	0.208
Changes of cholesterol (mg/dl)	21.09 (5.7)	12.6 (5.3)	0.290	19.9 (6.10)	13.7 (5.6)	0.482
Changes of ALT (U/L)	2.33 (4.58)	−2.77 (4.2)	0.420	−1.36 (4.6)	0.39 (4.28)	0.793
Changes of AST (U/L)	1.44 (3.3)	−1.12 (3.1)	0.736	−1.76 (3.35)	2.62 (3.08)	0.365
Changes of glucose (mg/dl)	12.0 (8.37)	1.09 (8.1)	0.368	8.30 (9.26)	4.28 (8.50)	0.763
Changes of insulin (μU/ml)	3.06 (2.5)	0.69 (2.3)	0.479	2.79 (2.72)	0.84 (2.45)	0.681
Changes of HOMA-IR	24.7 (17.7)	−2.40 (16.1)	0.267	21.7 (19.1)	−0.02 (17.1)	0.427
Changes of total muscle mass (kg)	−0.24 (0.3)	−0.33 (0.2)	0.832	−1.76 (3.35)	2.62 (3.08)	0.365
Changes of skeletal muscle (kg)	−0.19 (0.1)	−0.15 (0.1)	0.895	−0.30 (0.20)	−0.06 (0.19)	0.424

Median (interquartile range) for non-normally distributed variables. Covariates appearing in the model are evaluated for a change in value of body weight = −0.165 kg, and a change in value of caloric intake = −283.3 kcal. HOMA-IR, homeostasis model assessment – insulin resistance, *p < 0.05 by t-test and chi-square test.

### Intraobserver and intraclass correlation for MRI-PDFF

The intraobserver intraclass correlation coefficient (ICC) was 0.995 and the interobserver ICC was 0.994 for measurement of abdominal VFA. The difference in values measured twice by 1 reader was 10.6 cm^2^ and the difference measured by 2 readers was 12.8 cm^2^. The intra- and interobserver ICC was 0.999 for MRI-PDFF. The difference in values between 2 readers was 0.19% on average and the standard deviation was 0.2%. The difference range in values measured twice by one reader was 0–0.7% while the average difference of values was 0.23%, and the standard deviation was 0.16%.

### Liver stiffness

Changes in liver stiffness score measured with ultrasound (FibroScan^®^) were not different between probiotic (9.65 ± 16.2 kPa vs. 6.17 ± 2.5 kPa, p = 0.221) and placebo groups (6.76 ± 3.4 kPa vs. 6.18 ± 2.8 kPa, p = 0.142). Twenty-four patients in the probiotic group and 26 in the placebo group had acceptable results (success rate >60%, with an interquartile range/median value < 30%) on transient elastography.

### Liver enzyme, lipid profile, homeostatic model assessment of insulin resistance (HOMA-IR), and cytokine values

Total cholesterol (p < 0.001), triglyceride (p = 0.02), and tumor necrosis factor (TNF)-α (p = 0.007) levels decreased relative to baseline in the probiotic group (Table 4). The probiotic group also showed a greater decrease in triglyceride concentration (mean difference: −34.0 mg/dl, p = 0.0033) than the placebo group. ALT, AST, glucose, insulin, HOMA, and lipopolysaccharide values did not change in either group.

**Table 4:** Changes in biochemical variables between probiotic- and placebo-treated patients.

	Probiotic (n = 30)			Placebo (n = 35)			Difference (p value)
	Baseline	Posttreatment	*P	Baseline	Posttreatment	*P	
Cholesterol (mg/dl)	**201.3 ± 33.8**	**179.8 (167.5)**	**<0.001**	**196.7 ± 40.8**	**184.3 ± 36.8**	**0.03**	9.15 (0.240)
Triglyceride (mg/dl)	**187.4 (152)**	**150.4 ± 77.5**	**0.02**	172.8 (140)	177.3 (139)	0.53	**−34.0 (0.033)**
HDL-CROL (mg/dl)	**46.6 (43)**	**42.9 (39.5)**	**0.007**	47.37 (48)	44.7 (45)	0.09	<0.001 (0.707)
Glucose (mg/dl)	112.7 (92.5)	99.4 (94)	0.38	100.0 (93)	100.1(94)	0.63	−3.5 (0.333)
AST (U/L)	33.6 (27)	31.7 (28.5)	0.32	32.0 (23)	32.5 (22)	0.28	−1 (0.644)
ALT (U/L)	41.1 (31.5)	38.8 (35)	0.81	41.7 (28)	44.5 (23)	0.92	−1.5 (0.827)
Insulin (μU/ml)	17.1 (10.15)	14.1 (10.62)	0.67	12.8 (10.2)	12.1 (8.9)	0.61	−0.23 (0.937)
HOMA-IR	93.2 (47.14)	68.6 (46.11)	0.44	56.2 (46.4)	59.4 (35.4)	0.95	1.47 (0.614)
IL-6 (pg/ml)	1.45 (0.96)	1.64(1.17)	0.79	**2.25 (1.21)**	**1.22 (1.05)**	**0.04**	0.11 (0.157)
TNF-α (pg/ml)	**8.51 (7.69)**	**6.99 ± 2.92**	**0.011**	**9.20 (6.47)**	**6.99 (5.79)**	**0.01**	0.345 (0.757)
LPS (ng/ml)	0.06 (0.03)	0.07 (0.03)	0.65	0.05 (0.02)	0.04 (0.06)	0.50	<0.001 (0.928)

Data are expressed as median (interquartile range) with p values from Wilcoxon signed rank test or mean difference with p value in parentheses.

HDL-CROL, high-density lipoprotein cholesterol; AST, aspartate transaminase; ALT, alanine transaminase; HOMA-IR, homeostasis model assessment – insulin resistance; IL-6, interleukin-6; TNF-α, tumor necrosis factor-α; LPS, lipopolysaccharide; *p < 0.05 by paired t test.

### Change in gut microbiota

*L. acidophilus*, *L. rhamnosus*, *P. pentosaceus, B. lactis*, and *B. breve*, but not *L. paracasei*, were increased in the probiotic mixture group. However, *P. pentosaceus* was only increased in the placebo group (Supplementary Table 1). *Eubacterium, Fusicatenibacter dorea* (Operational Taxonomic Unit [OTU] 195044), *Oscillibacter*, and *Faecalibacterium* were significantly increased in patients with decreased BMI, and *Oscillibacter, Faecalibacterium, Ruminococcus*, and *Dorea* (OTU 1076587) were significantly increased in patients with increased BMI (Supplementary Table 2).

### Life style control and adverse events

Compared to baseline, there was no difference in the amount of dietary intake and exercise before and after treatment for 12 weeks in either the probiotic or placebo group (Supplementary Tables 3 and 4), although caloric intake at 12 weeks was decreased in the placebo group. Total energy intake decreased by 136 kcal in the probiotic group and 429 kcal in the placebo group during the intervention period, but there was no statistical difference between the groups (Supplementary Table 3). There was no change in the composition of macronutrient intake (carbohydrate, fat, and protein) between baseline and the end of intervention. There was no difference in physical activity between the probiotic and placebo groups (Supplementary Table 4). No difference in the incidence of adverse effects was seen between the groups, but 1 patient in the placebo group died of interstitial pneumonia. However, no correlation with drug treatment was found.

## Discussion

These results showed that 12 weeks of probiotic treatment significantly reduced hepatic fat and BMI in obese NAFLD patients. To the best of our knowledge, this is the first randomized controlled trial to examine the effects of probiotics on hepatic fat content and hepatic fibrosis using a reliable technique.

Two randomized controlled trials examining the effects of probiotics in NAFLD have previously been published^5,9^. The primary endpoint of both studies was liver ALT levels; changes in hepatic fat content and fibrosis were not evaluated. Moreover, changes in intestinal microbiota were not evaluated after probiotic treatment in either of these studies.

We administered a mixture of probiotics (*L. acidophilus, L. rhamnosus, L. paracasei, P. pentosaceus, B. lactis*, and *B. breve*) for 12 weeks. To select probiotic strains, we performed *in vitro* and *in vivo* studies using 3T3-L1 cells and a high-fat diet animal model, respectively (data not shown). We screened more than 100 strains using our own platform, and finally selected 6 probiotic bacterial species. All but *L. paracasei* increased in stool samples, based on quantitative polymerase chain reaction testing. However, there was no direct association between these 6 probiotic species and clinical parameters (decrease in body weight and hepatic fat). *Agathobaculum*, *Dorea* (OTU 527923), *Dorea* (OTU 195044), *Blautia*, *Ruminococcus*, and *Dorea* (OTU 470168) were significantly increased after probiotic treatment, but not in the placebo group, and were associated with a decreased hepatic FF. *Eubacterium*, *Fusicatenibacter*, *Dorea* (OTU 195044), *Oscillibacter*, and *Faecalibacterium* were increased after probiotic treatment, but not in the placebo group, and were associated with decreased BMI. *Dorea* (OTU 195044) showed a positive correlation with improvement in NAFLD and obesity (Table 5). These data suggested that some probiotic agents may have a priming or triggering effect in the correction of dysbiosis rather than a direct effect related to decreased hepatic fat and weight loss, in association with a dramatic increase in the amount of probiotic species in the intestinal bacterial community. Probiotic species inhibit pathogens by producing lactic acids, promoting innate and adaptive molecular immunity, and causing changes in the host gut environment^14,15^. This may contribute to modulation of the intestinal flora through an increase in specific bacteria such as *Agathobaculum*, *Dorea* (OTU 527923), *Dorea* (OTU 195044), *Blautia*, *Ruminococcus*, and *Dorea* (OTU 470168). Future research should investigate the interaction between probiotic species and these strains.

**Table 5:** Changes in gut microbiome related to fatty liver decrease or increase by probiotic intervention.

	Decreased liver fat (FF ≤ −1.7%)										Increased liver fat (FF ≥ 1.7%)									
	Probiotic Baseline		Probiotic 12 weeks		p value	Placebo Baseline		Placebo 12 weeks		p value	Probiotic Baseline		Probiotic 12 weeks		p value	Placebo Baseline		Placebo 12 weeks		p value
	Mean	SE	Mean	SE		Mean	SE	Mean	SE		Mean	SE	Mean	SE		Mean	SE	Mean	SE	
*Agathobaculum*	564.8	319.9	2214	534.5	**0.03**	1844	1104	1899	1101	0.93	2380	1146	3167	1023	0.48	2662	1774	6520	2386	0.25
*Dorea* (OTU 527923)	587	119	1680	307.2	**0.01**	1186	487.3	1461	503.3	0.74	1306	572	2006	544.6	0.38	1201	641.5	3459	707.3	0.12
*Dorea* (OTU 195044)	363.1	60.67	760.1	127	**0.01**	560	145.4	660	237.3	0.68	725.5	91.93	1366	608.4	0.39	960.8	309.1	1327	405.3	0.52
*Blautia*	161.9	65.52	491.7	148	**0.03**	543.2	124.4	647.6	229.6	0.66	440.8	229.5	784	376.3	0.26	398	160.5	1598	611.4	**0.02**
*Clostridium*	148.9	57.16	381.4	149.3	0.20	72	24.16	625.4	148.1	**0.02**	323.5	314.5	2454	2300	0.44	134.8	47.21	1304	1274	0.41
*Ruminococcus*	55.8	13.9	345.3	98.52	**0.01**	256.6	98.44	158.6	61.83	0.27	241.3	124.6	627.8	251.3	0.16	88.5	42.85	248.5	85.52	**0.03**
*Clostridium* (OTU 363710)	76.9	19.7	152.6	50.82	0.22	43.2	15.3	239.2	41.95	**0.01**	345.5	216	998.3	906.7	0.44	52	18.69	458.5	428.9	0.40
*Dorea* (OTU 470168)	50.5	15.75	267.5	82.79	**0.02**	128.4	60.84	108.2	41.57	0.78	209	114.2	158	34.12	0.72	51.5	17.83	227.3	59.57	**0.03**

Data are expressed as mean with P values from paired t-test between 0 and 12-week samples. SE, standard error.

Some findings of this study conflict with those of previous reports^16,17^. A recent study showed that *Ruminococcus, Blautia*, and *Dorea* were increased in pediatric nonalcoholic steatohepatitis (NASH) patients, relative to those in controls, and *Oscillospira*, Rikenellaceae*, Parabacteroides, Bacteroides fragilis, Sutterella*, and Lachnospiraceae were increased in NAFLD^17^. These results were similar to those in a subsequent “meta-omics” study in adult NASH patients, suggesting that *Bacteroides* and *Ruminococcus* were independently associated with NASH and significant fibrosis, respectively^16^. However, Zhu *et al*. reported that *Blautia*, *Coprococcus*, and *Ruminococcus* were significantly decreased in NASH patients^1^. These data suggest that environmental factors and age also affect changes in the microbiome. In addition, different species/subspecies may have a variable range of functions. *Faecalibacterium prausnitzii* is an anti-inflammatory commensal agent that is associated with obesity. Foditsch *et al*. and Balamurugan *et al*. reported that *F. prausnitzii* was associated with weight gain^18,19^. *F. prausnitzii* phylotypes are important in homeostasis in humans^20^. In this study, increases in 2 different strains of *Dorea* (OTU 527923 and 195044) were associated with decreased hepatic FF and BMI, and an increase in 1 strain of *Dorea* (OTU 1076587) was associated with weight gain. This suggests a wide range of effects associated with the same genus, depending on the species. Whether the increase in these strains is secondary to correction of intestinal dysbiosis or directly associated with fatty liver and obesity requires further research.

This study used MRI-PDFF, which is widely used in research on NAFLD, to quantify IHF^21,22^. This technique can accurately measure the distribution of IHF across the liver in seconds, and similar to magnetic resonance spectroscopy is considered very accurate. The decrease in IHF was 40.0% and 17.1% in the probiotic and placebo groups, respectively. There is no consensus on cut-off values for defining a change in hepatic fat. We assumed that a change in the MRI-PDFF value of more than 1.7% was significant. Nouredin *et al*. used MRI-PDFF to show that the actual change was 1%^23^. We applied stricter criteria by assuming that the changes in IHF measured with MRI-PDFF in several other studies represented actual tissue changes if the values were 1.6–1.8%^24–26^.

Patients received exercise and diet instruction. Caloric intake was slightly reduced in both groups, but no statistical significance was observed. Moreover, a difference in the number of dietary calories was not associated with a decrease in hepatic fat in the probiotic group. The association between diet and hepatic fat was thought to be small in this study. There was no difference in physical activity level between the 2 groups.

This study had some limitations. First, IHF was measured accurately using MRI-PDFF, but whether hepatic inflammation improved could not be determined. For this determination, liver biopsy was needed, but could not be performed. Second, IHF measured with MRI-PDFF in the probiotic group was decreased, while the controlled attenuation parameter (CAP) measured with transient elastography was not decreased. CAP is useful for measuring hepatic steatosis. However, MRI-PDFF is superior to CAP in diagnosing and measuring steatosis in patients with fatty liver^27^. Moreover, while baseline CAP and MRI-PDFF values are strongly correlated in assessments of hepatic steatosis, a weak correlation has been observed between CAP changes after treatment and intrahepatic fat changes (%) using MRI-PDFF in a longitudinal setting^27,28^. CAP measurement was performed with the M probe of a FibroScan instrument, but not the XL probe. Patients with BMI ≥ 28 kg/m^2^ should be assessed with an XL probe to reduce scan failures and to increase the reliability of hepatic steatosis measurement^29^. Eight patients in this study were excluded owing to unreliable measurement results^29,30^. Because of the small sample size, further large-scale investigation is needed; moreover, 12 weeks is insufficient for evaluation of hepatic fibrosis. More convincing MR elastography or liver biopsy and long-term follow-up data are needed. In addition, as the current microbiome database and 16S rRNA sequencing are limited in the ability to distinguish among gut bacteria when more than 1,000 species are present, culturomics and whole-genome sequencing will be necessary to identify specific bacteria associated with disease and to investigate the association between probiotic and other bacteria.

This study showed that administration of probiotics in obese patients with NAFLD for 12 weeks was effective in reducing triglycerides as well as IHF. However, with adjustment for weight changes, the IHF level was not different between treated and control groups. This means that a decrease in hepatic fat is mainly due to changes in body weight. However, the exact mechanism is not known. Further studies involving an analysis of intestinal flora and the metabolomics of intestinal bacteria are expected to identify the exact role of probiotics in patients with NAFLD.

## Materials and Methods

### Study design

This study was designed as an investigator-initiated, randomized, double-blinded, placebo-controlled trial with allocation concealment, to evaluate changes in abdominal VFA and IHF on MRI-PDFF after administration of a probiotic mixture to obese adults for 12 weeks. A probiotic mixture of *L. acidophilus* CBT LA1, *L. rhamnosus* CBT LR5 isolated from Korean human feces, *L. paracasei* CBT LPC5 isolated from Korean fermented food (jeotgal), *P. pentosaceus* CBT SL4 isolated from a Korean fermented vegetable product (kimchi), *B. lactis* CBT BL3, and *B. breve* CBT BR3 isolated from Korean infant feces was used. The probiotic mixture contained 10^9^ cfu/1.4 g. The placebo was composed of dextran, maltodextrin, lemon flavor, and Mg stearate. The study protocol was approved by the Hanyang University Hospital Institutional Review Board and registered with the Clinical Research Information Service (KCT0001588, https://cris.nih.go.kr; date of registration: 04/08/2015). Written informed consent was obtained from each participant prior to enrolment. All methods were performed in accordance with relevant guidelines and regulations.

### Inclusion criteria

This study targeted patients who visited the department of gastroenterology of a single hospital. The study included men and women aged between 19 and 75 years, with BMI ≥ 25 kg/m^2^ and mean hepatic MRI-PDFF value ≥ 5.0%.

### Exclusion criteria

Patients with 1 or more of the following conditions were excluded from this study: men and women who drank an average ≥210 g/week and ≥140 g/week, respectively, for the prior 2 years; those with ≥10% weight loss within 6 months; those on a diet or exercise program within 3 months after screening that could influence the results of the clinical trial; those with comorbidities such as a positive hepatitis B surface antigen test, positive hepatitis C antibody test, or eating disorders (anorexia, bulimia); those who were treated with diuretics or drugs that could affect absorption, metabolism, and excretion; those who were treated with amphetamines, cyproheptadine, phenothiazines, appetite suppressants, or appetite stimulants; and those who took probiotics within 2 weeks before screening.

### Randomization and allocation concealment

Block randomization was used to create a 1:1 probiotic:placebo group ratio, while random allocation codes were created using a computer program (Random Application Software 2.0). A pharmacist who was not involved in this study was in charge of randomization. The probiotic and placebo group allocations were delivered in packages with an identical appearance and randomly-allocated numbers, which allowed the principal investigator, sub-investigators, pharmacist, clinical trial team, and study subjects to be blinded. Sealed opaque bags with sequential numbers were used for adequate allocation concealment. The randomization schedule was kept sealed until the analysis of results after the trial was completed or in the event of an emergency.

### Primary and secondary outcomes

The primary endpoints were changes in VFA and IHF at the 12th week compared to baseline as measured on MRI. The secondary endpoints included changes in weight, BMI, and body fat at the 12th week compared to baseline as measured with bioimpedance analysis, and changes in biochemical indicators at the 12th week compared to those at baseline.

### Sample size calculation

No prior study has assessed changes in IHF after administration of probiotics, but 1 study found a 6% decrease in IHF using colesevelam^31^. The significance level was 0.05 using a 2-tailed test, with a statistical power of 90%, based on a minimum requirement of 22 subjects in each group (a total of 44 for 1:1 allocation). A study using *L. gasseri* SBT2055 (LG2055) showed a 9.2-cm^2^ reduction in VFA (study group: −9.6 cm^2^, placebo group: −0.4 cm^2^), with a standard deviation of 12.8 cm^2,32^. Based on the above considerations, the significance level was set at 0.05, with 80% statistical power and a minimum requirement of 34 subjects in each group (a total of 68 for 1:1 allocation). Therefore, this study included a total of 68 patients.

### Hepatic fat quantification using MRI-PDFF

The FF map for each patient was analyzed by a radiologist with 5 years of experience, using the Philips IntelliSpace Portal (Philips, Best, The Netherlands); clinical information and MRI scanning sequences were blinded. Three 100-mm^2^ circular regions of interest (ROIs) with no overlap were located in Couinaud’s liver segments by avoiding large vessels, ducts, and image artifacts to obtain a total of 24 ROIs. Each ROI was placed to visually match as much as possible the location of adjacent vessels in 2 MRI scans (at baseline and after treatment). The average of 3 values from each liver segment was calculated.

### Intra- and inter-observer variability for MRI-PDFF and measurement of visceral fat

Since patients with high average IHF values were estimated to have greater variability than those with low average values, a total of 30 patients were selected, including 10 with <10% FF measured using MRI-PDFF, 10 with 10–20%, and 10 with ≥20%. To assess intra-observer agreement, the same radiologist measured MRI-PDFF (mean FF of the whole liver) and abdominal VFA using the MRI results of these 30 patients on a monthly basis; then, a second radiologist measured the MRI-PDFF (mean FF of the whole liver) and abdominal VFA again in the same manner. Patient information and examination sequences were all blinded.

### Transient elastography

Subjects were instructed to remain in supine position with the right hand above the head. The probe was placed perpendicular to the intercostal skin surface over the right side of the liver for measurement, and the median value was automatically calculated from at least 10 measurements. A FibroScan^®^ (EchoSens^®^, Paris, France) ultrasound device and an M probe were used. Readings with success rates >60% were considered significant and those with interquartile range (IQR) > 30% were excluded from data analysis^33^.

### Diet diary and exercise education

Subjects were instructed to record the average weekly or daily intake and the amount of intake per serving by food group. The subjects then underwent dietary evaluation by consulting with the investigator using the diet diary recorded at screening points, and were educated on appropriate daily nutritional intake and exercise. Compliance with diet and exercise were evaluated with a 5-point Likert scale. Higher scores indicated greater compliance with diet and exercise (1 = Completely Failed, 2 = Failed, 3 = Satisfactory, 4 = Good, 5 = Excellent). The diet diary and exercise program were evaluated using a validated questionnaire from the Korea National Health and Nutrition Examination Survey.

### Biochemical tests

The following were assessed after at least an 8-hour fast: Na, K, Cl, creatinine, blood urea nitrogen, total cholesterol, triglycerides, high-density lipoprotein cholesterol, fasting glucose, AST, ALT, interleukin-6, TNF-α, C-reactive protein, lipopolysaccharide, low-density lipoprotein cholesterol, and fasting insulin.

### Statistical analysis

Per-protocol analysis was implemented in the 65 of 68 patients who completed 3 months of the study. SPSS software (version 13.0; SPSS Inc., Chicago, IL, USA) was used for statistical analysis. Changes in VFA and IHF before and after measurement were analyzed using a paired t-test. The normality of data was judged using the Shapiro-Wilk test. Data following a normal distribution were analyzed using Student’s t-test and expressed as averages and standard deviations. Data not following a normal distribution were analyzed using the Wilcoxon rank-sum test and expressed as medians and IQRs. Intra- and interobserver variability of MRI-PDFF and abdominal VFA measurements were evaluated using ICCs.

### Fecal sample preparation

Fecal samples were collected at 0 and 12 weeks. Samples were frozen at −80 °C until DNA was extracted. Fecal DNA was extracted using a commercial FastDNA Spin Kit for soil DNA extraction kits according to the manufacturer’s instructions (MP Biomedicals, Santa Ana, CA, USA). Nano-drop and Qubit dsDNA BR assay kit (Thermo Fisher Scientific, Carlsbad, CA, USA) were used to measure the DNA concentration for NGS. The bacterial 16S rRNA gene (V4–V5 region) was amplified and purified using AMPure XP magnetic beads (Beckman Coulter, Brea, CA, USA). The purified products were mixed to generate a 4-nM library pool. The library pool was sequenced with 250-bp paired-end reads on the MiSeq platform (Illumina, San Diego, CA, USA).

### Analysis of gut microbiota data

Raw sequencing reads obtained from the MiSeq platform were imported into the CLC Genomics Workbench (Qiagen) for bioinformatic analysis. The Operational Taxonomic Units (OTUs) clustering module and NGS Core Tools were used for quality control. The OTUs were picked by mapping sequences against the Greengenes database (ver.13_5) and clustered at 97% identity. The OTUs were aligned using Multiple Sequence Comparison by Log-Expectation and were used to construct a ‘Maximum Likelihood phylogenetic tree’ followed by diversity analyses. GraphPad Prism software was used to compare the composition of gut microbiota.

## Supplementary information

Supplement Table 1
