# Supplementary material for: Randomized, Double-blind, Placebo-controlled Study of a Multispecies Probiotic Mixture in Nonalcoholic Fatty Liver Disease
Source: Sci Rep. 2019 Apr 5;9:5688. doi: 10.1038/s41598-019-42059-3 (PMC6450966; doi:10.1038/s41598-019-42059-3)
Supplement: Supplementary file 1 — Supplement Table 1 [file 41598_2019_42059_MOESM1_ESM.docx]

**A Randomized, Double-blind, Placebo-controlled Study of a Multispecies Probiotic Mixture in Non-alcoholic fatty liver disease**

Sang Bong Ahn^1^, Dae Won Jun^2*^, Bo-Kyeong Kang^3*^, Jong Hyun Lim^4^, Sanghyun Lim^4^ and Myung-Jun Chung^4^

^1^Department of Internal Medicine, Eulji University School of Medicine, ^2^Department of Internal Medicine and ^3^Department of Radiology, Hanyang University School of Medicine, Hanyang University Medical Center, Seoul, ^4^R&D center Microbiome, Cell Biotech, Gyeongi, Korea

**Supplementary Tables**

Supplementary Table 1. Change of Microbiota : Probiotics versus placebo

|  | **Probiotics (n=29)** | | | **Placebo (n=31)** | | | **Difference**  **(*P* value)** |
| --- | --- | --- | --- | --- | --- | --- | --- |
|  | Baseline | Posttreatment | *P | Baseline | Posttreatment | *P |  |
| *Lactobacillus acidophilus* | 6.0x10^5^ | 5.4x10^9^ | **0.014** | 1.1x10^7^ | 1.1x10^5^ | 0.481 | 4537.3(0.4367) |
| *Lactobacillus rhamnosus* | 8.7x10^4^ | 2.1x10^6^ | **<0.001** | 2.5x10^4^ | 3.1x10^5^ | 0.096 | 129319.3(0.0727) |
| *Lactobacillus paracasei* | 9.2x10^5^ | 1.4x10^9^ | 0.127 | 1.5x10^7^ | 7.3x10^5^ | 0.505 | -2687.18(0.7479) |
| *Pediococcus pentosaceus* | 8.0x10^4^ | 2.9x10^8^ | **0.004** | **1.0x10^7^** | **1.4x10^6^** | **0.019** | -5738.2(0.8303) |
| *Bifidobacterium lactis* | 3.5x10^6^ | 1.3x10^8^ | **<0.001** | 1.1x10^7^ | 1.5x10^7^ | 0.142 | 3047607(0.3407) |
| *Bifidobacterium breve* | 3.5x10^5^ | 1.0x10^6^ | **0.042** | 6.5x10^5^ | 4.1x10^5^ | 0.233 | -3406.06(0.8829) |
| *Firmicutes* | 1.3x10^10^ | 9.4x10^9^ | 0.503 | 1.9x10^10^ | 9.9x10^9^ | 0.183 | -3.63 x10^9 (^0.5113) |
| *Bacteroidetes* | 2.5x10^9^ | 5.0x10^9^ | 0.080 | 2.8x10^9^ | 5.2x10^9^ | 0.108 | -6.23 x10^8 (^0.7888) |
| *B/F ratio* | 0.46±0.81 | 27.29±142.7 | **0.045** | 0.27±0.44 | 1.53±5.00 | 0.065 | 0.113(0.5827) |

B/F ratio: Bacteroidetes/ Firmicutes ratio. **P*<0.05 by Wilcoxon log rank test

Supplementary Table 2. Changes in gut microbiome related to BMI improvement or worse by probiotics intervention

|  | **Decreased BMI** | | | | | | | | | | **Increased BMI** | | | | | | | | | |
| --- | --- | --- | --- | --- | --- | --- | --- | --- | --- | --- | --- | --- | --- | --- | --- | --- | --- | --- | --- | --- |
|  | Probiotics  Baseline | | Probiotics  12 weeks | | *P* value | Placebo  Baseline | | Placebo  12 weeks | | *P* value | Probiotics  Baseline | | Probiotics  12 weeks | | *P* value | Placebo  Baseline | | Placebo  12 weeks | | *P* value |
|  | Mean | SE | Mean | SE |  | Mean | SE | Mean | SE |  | Mean | SE | Mean | SE |  | Mean | SE | Mean | SE |  |
| *Eubacterium* | 861.6 | 228 | 1323 | 368.9 | **0.04** | 2301 | 512.2 | 2228 | 762.4 | 0.91 | 507 | 306.5 | 1346 | 324.5 | 0.12 | 1957 | 613.2 | 3100 | 1203 | 0.13 |
| *Fusicatenibacter* | 883.7 | 267.5 | 1509 | 263.6 | **0.02** | 1417 | 496.4 | 1839 | 897.8 | 0.77 | 655 | 244.9 | 2172 | 863.2 | 0.10 | 1200 | 395.4 | 2493 | 595.3 | 0.13 |
| *Dorea*  (OTU 195044) | 431 | 109.7 | 785.4 | 146.5 | **0.01** | 886 | 350.8 | 487.7 | 57.81 | 0.40 | 468 | 92.4 | 1276 | 489.1 | 0.17 | 774.5 | 181.8 | 928.4 | 259.9 | 0.59 |
| *Oscillibacter* | 949.2 | 532.2 | 1773 | 481.2 | **0.02** | 6168 | 5058 | 949.7 | 275.6 | 0.40 | 635.8 | 217.2 | 2870 | 977.9 | **0.04** | 2352 | 1095 | 6123 | 2216 | 0.06 |
| *Faecalibacterium* | 598.4 | 237 | 1420 | 380.5 | **0.02** | 2574 | 1252 | 814 | 264.8 | 0.30 | 606.8 | 312 | 2230 | 652.8 | **0.02** | 983.9 | 384 | 4977 | 1487 | **0.01** |
| *Ruminococcus* | 160.3 | 62.51 | 290 | 84.3 | 0.08 | 2574 | 1262 | 814 | 264.8 | 0.53 | 101.8 | 43.38 | 685 | 161.6 | **0.01** | 983.9 | 384 | 4977 | 1487 | 0.46 |
| *Dorea*  (OTU 1076587) | 101.3 | 44.22 | 158.3 | 39.46 | 0.35 | 93.3 | 87.8 | 179.3 | 141.9 | 0.69 | 19.6 | 8.2 | 236.8 | 63.37 | **0.03** | 108 | 34 | 201.3 | 36.3 | 0.16 |
| *Faecalibacterium* (OTU 851865) | 171.6 | 93.11 | 606.1 | 263 | 0.09 | 805.7 | 501.1 | 290.7 | 124.9 | 0.36 | 142.4 | 49.44 | 1078 | 472.2 | 0.11 | 231.6 | 56.53 | 2199 | 847.1 | **0.05** |
| *Blautia* | 326.8 | 122 | 567.4 | 183.8 | 0.07 | 623 | 125.9 | 490 | 164.5 | 0.68 | 55.4 | 22.3 | 464.6 | 209.8 | 0.13 | 453.9 | 96.6 | 1107 | 249.7 | **0.03** |

Data are expressed as mean with *P* values from paired t test between 0 and 12 week samples. SE, standard error

Supplementary Table 3. Change of diet during treatment

|  | **Probiotics (n=30)** | | | | **Placebo (n=31)** | | | **Difference**  **(*P* value)** |
| --- | --- | --- | --- | --- | --- | --- | --- | --- |
|  | Baseline | Posttreatment | | *P | Baseline | Posttreatment | *P |  |
| Total calorie intake (Kcal) | 2147±845 | 2011±692.3 | | 0.343 | 2474.8(2266.1) | 2045.1(1882.7) | **0.004** | 331.86(0.1468) |
| Carbohydrate intake (g) | 336.9±138 | 316.7±120 | | 0.435 | 375.9(354.6) | 322.3(284.9) | 0.067 | 226.87(0.4922) |
| Fat intake (g) | 56.2(54.0) | 52.1±20.2 | | 0.465 | 68.99(56.93) | 52.9(48.04) | **0.0007** | 40.94(0.5089) |
| Vegetable fat (g) | 31.89(28.94) | 28.07±11.84 | | 0.183 | 37.89(33.42) | 29.1(25.13) | **0.001** | 3.325(0.1102) |
| Animal fat (g) | 24.26±11.63 | 24.03±11.06 | | 0.907 | 31.09(24.54) | 23.88(22.44) | **0.026** | 0.8605(0.5258) |
| Protein (g) | 80.47±32.72 | 75.99±25.33 | | 0.379 | 95.39(85.82) | 78.2(71.48) | **0.001** | 7.64(0.1326) |
| Vegetable protein (g) | 42.80±17.59 | 39.23±15.43 | | 0.219 | 47.43±18.07 | 39.22(34.87) | **0.004** | 11.24(0.2977) |
| Animal protein (g) | 37.67±17.59 | 36.75±15.79 | | 0.771 | 47.43(37.53) | 38.98(37.06) | **0.039** | -0.259(0.4759) |
| Fiber (g) | 25.81(24.86) | 25.59±10.94 | | 0.515 | 29.55(25.66) | 26.95(21.80) | 0.119 | 0.94(0.3913) |
| Vitamin A (μg) | 1211.03(1161.13) | 1092.79(998.85) | | 0.198 | 1339.3±646.5 | 1180.1(908.65) | 0.100 | 100.74(0.3101) |
| Retinol (μg) | 199.38(177.48) | | 202.04±110.27 | 0.745 | 264.22(223.7) | 177.2±91.6 | 0.00006 | 115.44(0.3228) |
| β carotene (μg) | 6075.52(5390.08) | | 5349.51(4753.40) | 0.228 | 6456.5±3539.6 | 6022.2(4726.7) | 0.334 | 613.01(0.5174) |
| Vitamin D (μg) | 4.98(4.10) | | 5.03(4.28) | 0.823 | 6.00(4.24) | 5.36(4.24) | 0.168 | 28.2125(0.9323) |
| Vitamin E (mg) | 20.76(20.50) | | 19.2±6.9 | 0.569 | 24.52±11.33 | 20.1(16.36) | 0.002 | 1.63(0.1505) |
| Vitamin K (μg) | 220.79±116.65 | | 193.98±68.51 | 0.063 | 254.9±124.1 | 207.7(157.6) | **0.004** | 33.61(0.2477) |
| Vitamin C (mg) | 116.93(105.74) | | 115.01±53.61 | 0.670 | 132.55±71 | 139.9(106.2) | 0.532 | 30.35(0.3292) |

Data are expressed as median (interquartile range) with P values from Wilcoxon signed rank test or mean difference with P value in parentheses. **P* < 0.05 by paired t-test.

Supplementary Table 4. Change of physical activity during treatment

|  | Probiotic group | Placebo group | **P* |
| --- | --- | --- | --- |
| Severe physical activity >10 min/week^1)^ (day) | 1.83 ± 1.3 | 1.56 ± 1.4 | 0.361 |
| Severe physical activity^1)^/ day (min) | 1.31 ± 2.4 | 2.03 ± 2.6 | 0.220 |
| Moderate physical activity >10min/week^2)^ (day) | 1.38 ± 0.8 | 1.88 ± 1.6 | 0.055 |
| Moderate physical activity^2)^ /day (min) | 1.75 ± 2.2 | 1.20 ± 2.2 | 0.358 |

1) Severe physical activity; Running, climbing, bike, fast swimming, football, basketball, jump rope, squash, singles tennis; 2) Moderate physical activity ; Slow swimming, doubles tennis, volleyball, Badminton, table tennis. **P* < 0.05 by t-test.
